# Exercise‐Related Out‐of‐Hospital Cardiac Arrest Among the General Population in the Era of Public‐Access Defibrillation: A Population‐Based Observation in Japan

**DOI:** 10.1161/JAHA.117.005786

**Published:** 2017-06-13

**Authors:** Kosuke Kiyohara, Chika Nishiyama, Takeyuki Kiguchi, Tatsuya Nishiuchi, Yasuyuki Hayashi, Taku Iwami, Tetsuhisa Kitamura

**Affiliations:** ^1^ Department of Public Health Tokyo Women's Medical University Tokyo Japan; ^2^ Department of Critical Care Nursing Kyoto University Graduate School of Human Health Science Kyoto Japan; ^3^ Kyoto University Health Service Kyoto Japan; ^4^ Department of Acute Medicine Kindai University Faculty of Medicine Osaka Japan; ^5^ Senri Critical Care Medical Center Osaka Saiseikai Senri Hospital Osaka Japan; ^6^ Division of Environmental Medicine and Population Sciences Department of Social and Environmental Medicine Graduate School of Medicine Osaka University Osaka Japan

**Keywords:** automated external defibrillator, cardiac arrest, cardiopulmonary resuscitation, exercise, Epidemiology, Exercise, Cardiopulmonary Arrest

## Abstract

**Background:**

Exercise can trigger sudden cardiac arrest. Early initiation of cardiopulmonary resuscitation and automated external defibrillator use by laypersons could maximize the survival rate following exercise‐related out‐of‐hospital cardiac arrest (OHCA).

**Methods and Results:**

OHCA data between 2005 and 2012 were obtained from a prospective population‐based OHCA registry in Osaka Prefecture. Patients with OHCA of presumed cardiac origin and occurring before emergency medical service personnel arrival were included. The incidence trends of exercise‐related OHCA over the 8‐year study period were assessed. Among patients with bystander‐witnessed, exercise‐related OHCA, the trends in the initiation of bystander cardiopulmonary resuscitation, public‐access defibrillation, and outcome were evaluated. The primary outcome was 1‐month survival with favorable neurological outcome, defined as cerebral performance category 1 or 2. During the study period, 0.7% of OHCAs of cardiac origin (222/31 030) were exercise related. The incidence of exercise‐related OHCA increased from 1.8 (per million population per year) in 2005 to 4.3 in 2012. Of these, 83.8% (186/222) were witnessed by bystanders. Among the patients with bystander‐witnessed, exercise‐related OHCA, the proportion that received bystander cardiopulmonary resuscitation (50.0% in 2005 and 86.2% in 2012) and public‐access defibrillation (7.1% in 2005 and 62.1% in 2012) significantly increased during the study period. Furthermore, the rate of 1‐month survival with favorable neurological outcome among these patients significantly improved (from 28.6% in 2005 to 58.6% in 2012).

**Conclusions:**

The incidence rate of exercise‐related OHCA was low in the study population. The increase in bystander cardiopulmonary resuscitation and public‐access defibrillation rates were associated with improved outcome among patients with bystander‐witnessed, exercise‐related OHCA.

**Clinical Perspective**

**What Is New?**

- This study evaluated the long‐term changes in the frequency of bystander cardiopulmonary resuscitation and public‐access defibrillation on the outcome of exercise‐related outof‐hospital cardiac arrest.

**What Are the Clinical Implications?**

- The incidence rate of exercise‐related outof‐hospital cardiac arrest in Osaka Prefecture has increased, although it remained low during the 8‐year study period.
- The significant increases in bystander cardiopulmonary resuscitation and public‐access defibrillation were associated with a substantial improvement in neurologically intact survival after exercise‐related outof‐hospital cardiac arrest.
- Neurologically‐intact survival following exercise‐related outof‐hospital cardiac arrest can be very high if bystanders recognize the cardiac arrest, phone the emergency response number, begin bystander cardiopulmonary resuscitation and use an automated external defibrillator before the arrival of emergency medical service personnel.

## Introduction

Although exercise plays an important role in preventing chronic heart disease and maintaining good health,^1^ recent studies have reported a transient rise in the incidence of acute myocardial infarction and sudden cardiac arrest during strenuous exercise.^2^, ^3^, ^4^ Consequently, improving bystander response and survival following exercise‐related cardiac arrest are critical public health issues. Out‐of‐hospital cardiac arrest (OHCA) during exercise is likely to be a witnessed event; therefore, early initiation of cardiopulmonary resuscitation (CPR) and automated external defibrillator (AED) use by bystanders could be critical in maximizing patient survival following cardiac arrest.^5^, ^6^, ^7^, ^8^

In Japan, AED use by nonmedical persons for patients with OHCA has been legal since July 2004. The number of public‐access AEDs has rapidly increased, with >360 000 devices located throughout Japan in 2012 (including 25 000 in Osaka Prefecture).^9^ Previous studies suggested that the nationwide dissemination of public‐access AEDs allowed early defibrillation by bystanders, leading to increased survival rates after OHCA.^10^, ^11^ Nevertheless, in the era of the public‐access defibrillation (PAD), we identified no published studies of the long‐term effects of bystander CPR and public‐access AED on the outcome of exercise‐related OHCA. Furthermore, improved understanding of the characteristics of patients with OHCA and prehospital factors related to outcome following OHCA during exercise could be instrumental in planning appropriate preventive strategies in the community setting.

The Utstein Osaka Project is a large prospective population‐based cohort study of patients with OHCA, covering Osaka Prefecture, the largest prefecture in western Japan, with ≈8.8 million inhabitants and spanning an area of 1892 km^2^.^12^ Using this registry, we investigated the trends in OHCA incidence rate, patient characteristics, prehospital interventions by bystanders, and outcomes of exercise‐related OHCA. To emphasize the epidemiological characteristics of exercise‐related OHCA, we compared the characteristics of patients with exercise‐related and non–exercise‐related OHCA to identify differences.

## Methods

### Study Design

Data registration of the Utstein Osaka Project was conducted according to the international Utstein Style guidelines, which are utilized worldwide as a standardized guideline for reporting outcomes of cardiac arrest.^13^, ^14^ Cardiac arrest is defined as the cessation of cardiac mechanical activity and confirmed by the absence of signs of circulation. The arrest was assumed to be of cardiac origin unless a noncardiac cause was confirmed; noncardiac causes include external causes (suffocation, hanging, fall, drowning, traffic injury, drug overuse, and unclassified external causes), respiratory disease, malignancy, or stroke.^13^, ^14^ According to the hospital medical records, noncardiac causes were defined clinically by the physicians in charge, in collaboration with emergency medical service (EMS) personnel. All survivors of OHCA were followed up for up to 1 month after the event by the EMS providers in charge, to assess survivor neurological outcome. Information from input data forms were transferred and integrated into the registration system at the Information Center for Emergency Medical Services of Osaka. The data were then checked by the investigators, and when incomplete data were identified, EMS personnel in charge were asked to complete the data forms.

### EMS System in Osaka

Detailed characteristics of the EMS system in Osaka Prefecture have been described elsewhere.^15^, ^16^ Briefly, in Osaka, there are 34 fire stations with emergency dispatch centers operating a 24‐hour EMS system. Of these, 32 stations are single‐tiered (paramedics only) and 2 are 2‐tiered (paramedics and physicians). Following a 119 call (the emergency telephone number for calling the ambulance service or fire department in Japan), the emergency dispatch center sends the nearest available ambulance to the call location. Protocols for care of those with OHCA are basically uniform among the EMS systems in Japan and follow the Japanese resuscitation guidelines. All EMS personnel perform CPR on victims, according to the Japanese CPR guidelines.^17^ Generally, prehospital termination of resuscitation by EMS personnel is not permitted because do‐not‐resuscitate orders (or living wills) are not permitted in Japan. Excluding cases of decapitation, incineration, decomposition, rigor mortis, or dependent cyanosis, EMS personnel attempt resuscitation for all patients with OHCA and transport the patients to hospitals and register them in the Utstein Osaka Project.

### Data Collection

We obtained the following data items from the registry for this study: age, sex, location of cardiac arrest, activities at the time of cardiac arrest, cardiac arrest witness, history of ischemic heart disease, ability to perform activities of daily living (ADLs) before cardiac arrest, first documented cardiac rhythm, dispatcher instruction, initiation of bystander CPR, PAD, time from collapse to patient contact with EMS personnel. Outcome data, including prehospital return of spontaneous circulation, 1‐month survival, and 1‐month neurological status after OHCA, were also obtained. The neurological status was assessed by the physician in charge, using the cerebral performance category scale: category 1, good performance; category 2, moderate disability; category 3, severe cerebral disability; category 4, coma/vegetative state; and category 5, death or brain death.^13^, ^14^

### Study Participants

OHCA data were obtained between January 1, 2005, and December 31, 2012. Patients who suffered from an OHCA of presumed cardiac origin were included. Patients for whom resuscitation by EMS personnel or bystanders was not attempted (cases of decapitation, incineration, decomposition, rigor mortis, or dependent cyanosis) and patients who had a cardiac arrest that was witnessed by EMS personnel were excluded from our analyses.

### Key Group Definition

In the Utstein Osaka Project, patient activities at the time of cardiac arrest were recorded by the EMS personnel in charge according to 6 categories (ie, *exercising, bathing, working, sleeping, unspecific activities*, and *unknown*) based on interviews with bystanders at the scene. Except for unknown cases, activity that could not be considered exercising, bathing, working, or sleeping, was labeled *unspecific activities* by the EMS personnel. In this study, OHCA cases associated with exercise at the time of arrest were labeled *exercise‐related* OHCA. The remaining cases were labeled *non–exercise‐related* OHCA.

### Outcome Measures

The primary outcome measure was 1‐month survival with favorable neurological outcome, defined as cerebral performance category 1 or 2.^13^, ^14^ The secondary outcome measures were prehospital return of spontaneous circulation and 1‐month survival after OHCA.

### Statistical Analysis

For all eligible patients, the annual incidence rates (per 1 million population) of exercise‐related and non–exercise‐related OHCA were calculated separately, and a Poisson regression model was applied for trend analysis. Subsequently, because witnessed cardiac arrest is a strong prognostic indicator, we conducted the following analyses focusing on bystander‐witnessed patients with OHCA. First, the changes in the proportions of patients receiving basic life support (bystander‐initiated CPR and PAD) and the outcome measures after an OHCA during the study period were evaluated using the Mantel‐Haenszel χ^2^ test of linear association. Second, the differences in patient characteristics between exercise‐related and non–exercise‐related OHCA were evaluated using the χ^2^ test for categorical variables and the unpaired *t* test or Mann–Whitney *U* test for continuous variables. Furthermore, a multivariate logistic regression model was used to investigate potential prehospital factors associated with 1‐month survival with favorable neurological outcome. The odds ratios (ORs) and associated 95% confidence intervals (CIs) were calculated. The explanatory variables considered in the analysis included activity at the time of OHCA (exercise and nonexercise), age, sex (male or female), location of arrest (home, street, sports facilities, or other), history of ischemic heart disease (yes or no), ADLs before OHCA (good or disability), first documented rhythm (*shockable* [ie, ventricular fibrillation or pulseless ventricular tachycardia] versus *nonshockable* [not ventricular fibrillation or pulseless ventricular tachycardia]), dispatcher instruction (yes or no), initiation of bystander CPR (yes or no), PAD (yes or no), time from collapse to contact with the patient by EMS personnel, and year in which the event occurred. All tests were 2‐tailed, and a *P* value <0.05 was considered statistically significant. Statistical analyses were conducted using the SPSS statistical package version 20.0J.

### Ethics

The study protocol was approved by the ethics committee of Osaka University with the assent of the EMS authorities of the local governments in Osaka Prefecture. The requirement for individual informed consent for the review of patient outcomes was waived by the Personal Information Protection Law and the national research ethics guidelines of Japan.

## Results

### Trends in Incidence Rate

**Figure 1:**
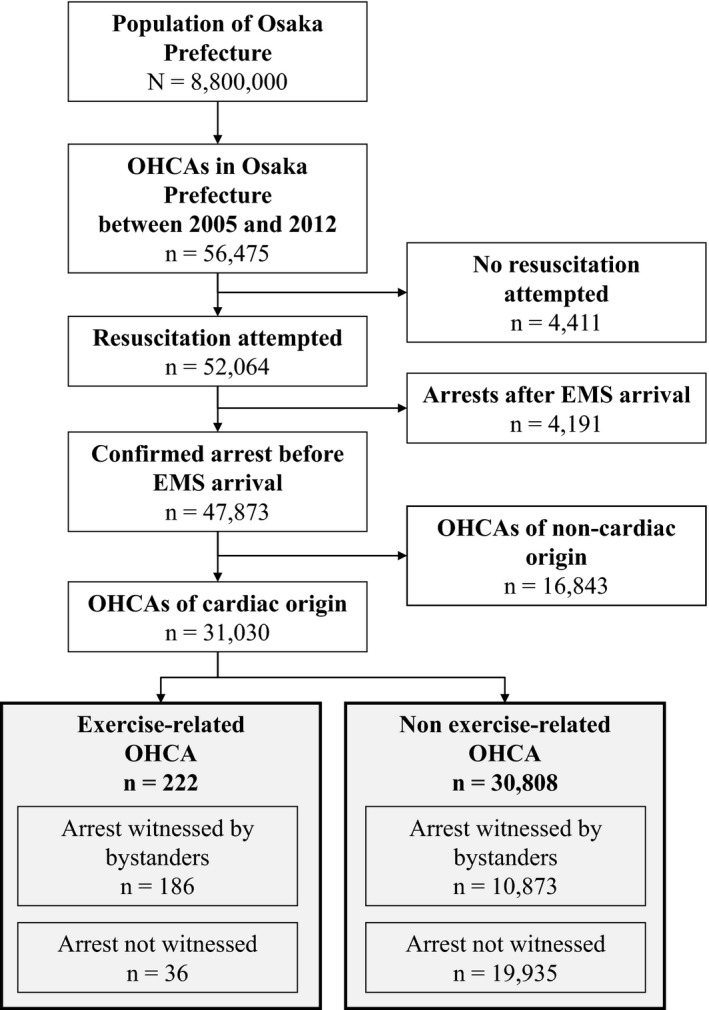
Selection of patients with out‐of‐hospital cardiac arrest (OHCA) of cardiac origin in Osaka Prefecture between January 1, 2005, and December 31, 2012. The patients were then sorted according to OHCA characterized as exercise‐related or non–exercise‐related. EMS indicates emergency medical service.

Figure 1 shows the selection of patients with OHCA of presumed cardiac origin in Osaka Prefecture during the 8‐year study period. A total of 31 030 OHCA patients of cardiac origin were recorded during the study period, and 0.7% (222/31 030) were exercise‐related OHCAs. Of these, 83.8% (186/222) were witnessed by bystanders. Table 1 shows the annual number and incidence of exercise‐related and non–exercise‐related OHCA of cardiac origin. Overall incidence rates of exercise‐related and non–exercise‐related OHCA were 3.1 and 435.2 per million population, respectively, during the study period. The annual incidence rates per million population significantly increased for both exercise‐related OHCA (from 1.8 in 2005 to 4.3 in 2012, *P*=0.034 for trend) and non–exercise‐related OHCA (from 360.0 in 2005 to 507.9 in 2012, *P*<0.001 for trend).

**Table 1:** Number of Patients and Incidence Rate of OHCA of Cardiac Origin in Osaka Prefecture Between 2005 and 2012

	Year								Total	*P* for Trend
	2005	2006	2007	2008	2009	2010	2011	2012		
Number of patients										
Exercise‐related OHCA	16	21	25	36	29	24	33	38	222	
Non–exercise‐related OHCA	3174	3279	3537	3960	3988	4089	4279	4502	30 808	
All OHCA	3190	3300	3562	3996	4017	4113	4312	4540	31 030	
Incidence rate per million population per y										
Exercise‐related OHCA	1.8	2.4	2.8	4.1	3.3	2.7	3.7	4.3	3.1	0.034
Non–exercise‐related OHCA	360.0	371.5	400.2	447.5	450.0	461.2	482.7	507.9	435.2	<0.001
All OHCA	361.8	373.8	403.0	451.5	453.3	463.9	486.4	512.2	438.3	<0.001

OHCA indicates out‐of‐hospital cardiac arrest.

### Patient Characteristics

**Figure 2:**
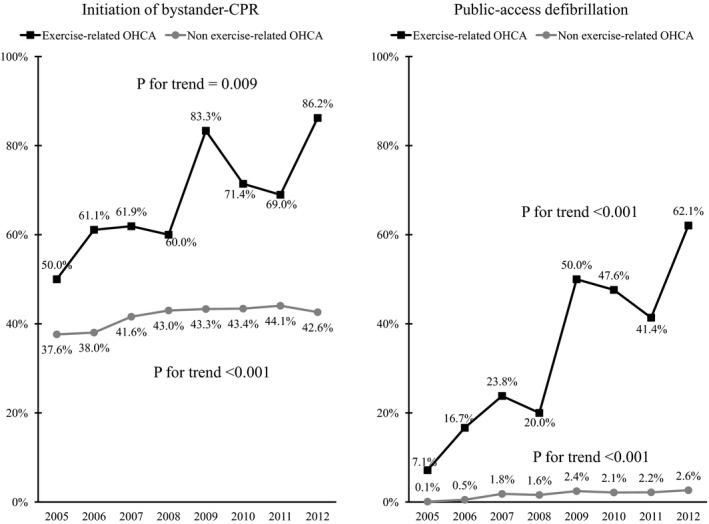
Trends in the proportion of patients with bystander‐witnessed out‐of‐hospital cardiac arrest (OHCA) of cardiac origin who received bystander‐initiated cardiopulmonary resuscitation and public access defibrillation between 2005 and 2012. CPR indicates cardiopulmonary resuscitation.

Figure 2 shows the trends in the proportion of patients sustaining a bystander‐witnessed OHCA of cardiac origin who received bystander‐initiated CPR and PAD during the study period. The proportion of patients with exercise‐related OHCA receiving bystander‐initiated CPR significantly increased during the study period (from 50.0% in 2005 to 86.2% in 2012, *P*=0.009 for trend). The proportion of those receiving PAD also significantly increased (from 7.1% in 2005 to 62.1% in 2012, *P*<0.001 for trend). Table 2 shows the patient characteristics of bystander‐witnessed OHCA of cardiac origin. Compared with non–exercise‐related OHCA, patients sustaining exercise‐related OHCA were significantly likely to be younger (median age: 63 versus 75 years) and male (85.5% versus 62.3%), with location of arrest in sports facilities (43.5% versus 0.2%), good ADLs (97.3% versus 66.7%), and ventricular fibrillation rhythm (36.6% versus 21.8%), and to receive bystander CPR (69.4% versus 41.9%), and PAD (36.0% versus 1.8%).

**Table 2:** Patients With Bystander‐Witnessed OHCA of Cardiac Origin: Comparison of Characteristics for Exercise‐Related and Non–Exercise‐Related Arrest

	Total (n=11 059)		Exercise‐related OHCA (n=186)		Non–exercise‐related OHCA (n=10 873)		*P* Value
Age, y, median (IQR)	75	(64–84)	63	(50–71)	75	(64–84)	<0.001
Age group, n, %							<0.001
Age <35 y	265	(2.4%)	26	(14.0%)	239	(2.2%)	
Age 35–69 y	3753	(33.9%)	105	(56.5%)	3648	(33.6%)	
Age ≥70 y	7041	(63.7%)	55	(29.6%)	6986	(64.3%)	
Men, n, %	6931	(62.7%)	159	(85.5%)	6772	(62.3%)	<0.001
Location of arrests, n, %							<0.001
Home	7161	(64.8%)	8	(4.3%)	7153	(65.8%)	
Street	751	(6.8%)	26	(14.0%)	725	(6.7%)	
Sports facilities	107	(1.0%)	81	(43.5%)	26	(0.2%)	
Other locations	3040	(27.5%)	71	(38.2%)	2969	(27.3%)	
History of ischemic heart disease, n, %	1569	(14.2%)	27	(14.5%)	1542	(14.2%)	0.897
Good activities of daily living before arrest, n, %	7430	(67.2%)	181	(97.3%)	7249	(66.7%)	<0.001
VF as first documented rhythm, n, %	2436	(22.0%)	68	(36.6%)	2368	(21.8%)	<0.001
Dispatcher instruction, n, %	3974	(35.9%)	49	(26.3%)	3925	(36.1%)	0.006
Initiation of bystander CPR, n, %	4689	(42.4%)	129	(69.4%)	4560	(41.9%)	<0.001
Shock by public‐access AED, n, %	259	(2.3%)	67	(36.0%)	192	(1.8%)	<0.001
Time from collapse to contact with patient by EMS, min, median (IQR)	9.0	(6–12)	9.0	(7–13)	9.0	(6–12)	0.075^c^
Time from collapse to shock by a public‐access AED, min,^a^ median (IQR)	5.0	(2–7)	4.5	(2–7)	5.0	(3–7)	0.265^d^
Time from collapse to first shock by EMS, min,^b^ median (IQR)	11.0	(8–16)	12.0	(9–16)	11.0	(8–16)	0.392^d^
Time from collapse to hospital arrival, min, median (IQR)	29.0	(24–37)	28.0	(23–35)	30.0	(24–37)	0.548^c^

AED indicates automated external defibrillator; CPR, cardiopulmonary resuscitation; EMS, emergency medical service; IQR, interquartile range; OHCA, out‐of‐hospital cardiac arrest; VF, ventricular fibrillation.

a Includes cases who were shocked by public‐access AED.

b Includes cases who were not shocked by public‐access AED.

c Unpaired t test was used.

d Mann–Whitney *U* test was used.

### Outcomes After OHCA

**Figure 3:**
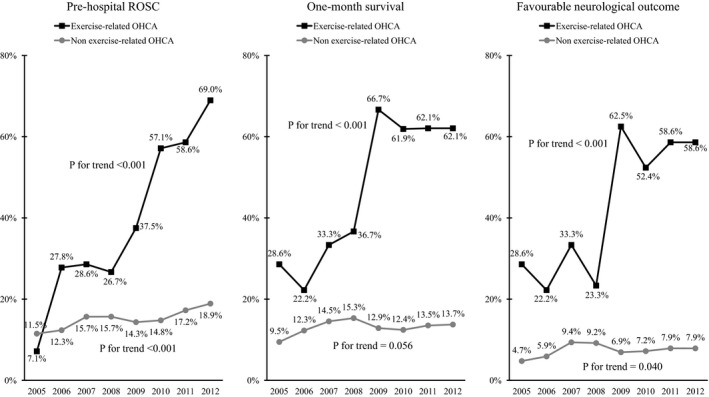
Trends in the outcomes after bystander‐witnessed out‐of‐hospital cardiac arrest (OHCA) of cardiac origin between 2005 and 2012. ROSC indicates return of spontaneous circulation.

Figure 3 shows the trends in the outcomes after bystander‐witnessed OHCA of cardiac origin during the study period. Among the exercise‐related patients with OHCA, prehospital return of spontaneous circulation, 1‐month survival, and 1‐month survival with favorable neurological outcome significantly improved during the study period (prehospital return of spontaneous circulation: 7.1% in 2005 and 69.0% in 2012, *P*<0.001 for trend; 1‐month survival: 28.6% in 2005 and 62.1% in 2012, *P*<0.001 for trend; 1‐month survival with favorable neurological outcome: 28.6% in 2005 and 58.6% in 2012, *P*<0.001 for trend).

Table 3 shows the association between prehospital factors and 1‐month survival with favorable neurological outcome after bystander‐witnessed OHCA of cardiac origin. Overall, the proportion of patients with favorable neurological outcome was 44.1% (82/186) in exercise‐related OHCA and 7.5% (812/10 867) in non–exercise‐related OHCA and was highest among OHCA occurring in sports facilities (50.5%, 54/107). In multivariable analysis, the following factors were associated with improved outcome: exercise‐related OHCA (adjusted OR: 1.85; 95% CI, 1.18–2.90), young age (1‐year incremental increase in age: adjusted OR: 0.98; 95% CI, 0.97–0.98), OHCA occurring on the street (adjusted OR: 1.50; 95% CI, 1.16–1.94), OHCA occurring in a sports facilities (adjusted OR: 1.84; 95% CI, 1.01–3.36), good ADLs before OHCA (adjusted OR: 2.03; 95% CI, 1.58–2.59), ventricular fibrillation rhythm as first documented rhythm (adjusted OR: 9.70; 95% CI, 8.01–11.74), bystander‐initiated CPR (adjusted OR: 1.38; 95% CI, 1.14–1.66), PAD (adjusted OR: 2.85; 95% CI, 2.06–3.95), and shorter EMS response time (1‐minute incremental increase in time from collapse to EMS contact: adjusted OR: 0.91; 95% CI, 0.89–0.93).

**Table 3:** Association Between Prehospital Factors and 1‐Month Survival With Favorable Neurological Outcome After Bystander‐Witnessed OHCA of Cardiac Origin

Factor (n=11 053)^a^	Favorable neurological outcome		Univariable analysis			Multivariable analysis		
	n/N	(%)	OR	(95% CI)	*P* Value	OR	(95% CI)	*P* Value
Activity at the time of OHCA								
Exercise	82/186	(44.1%)	9.76	(7.25–13.16)	<0.001	1.85	(1.18–2.9)	0.007
Nonexercise	812/10 867	(7.5%)	Ref.			Ref.		
Age (1‐y increment)	…		0.96	(0.96–0.96)	<0.001	0.98	(0.97–0.98)	<0.001
Sex								
Male	692/6927	(10.0%)	2.16	(1.83–2.54)	<0.001	0.93	(0.77–1.12)	0.417
Female	202/4126	(4.9%)	Ref.			Ref.		
Location of arrests								
Home	395/7158	(5.5%)	Ref.			Ref.		
Street	117/751	(15.6%)	3.16	(2.53–3.94)	<0.001	1.50	(1.16–1.94)	0.002
Sports facilities	54/107	(50.5%)	17.45	(11.78–25.83)	<0.001	1.84	(1.01–3.36)	0.045
Other locations	328/3037	(10.8%)	2.07	(1.78–2.42)	<0.001	1.14	(0.94–1.38)	0.190
History of ischemic heart disease								
Yes	159/1567	(10.1%)	1.35	(1.12–1.61)	0.001	1.17	(0.95–1.45)	0.135
No	735/9486	(7.7%)	Ref.			Ref.		
Good activities of daily living before arrest								
Good	803/7424	(10.8%)	4.72	(3.78–5.88)	<0.001	2.03	(1.58–2.59)	<0.001
Disability	91/3629	(2.5%)	ref.			ref.		
First documented rhythm								
VF	722/2629	(27.5%)	18.16	(15.27–21.61)	<0.001	9.70	(8.01–11.74)	<0.001
Non‐VF	172/8424	(2.0%)	ref.			ref.		
Dispatcher instruction								
Yes	300/3973	(7.6%)	0.89	(0.77––1.03)	0.121	0.96	(0.79–1.17)	0.687
No	594/7080	(8.4%)	ref.			ref.		
Initiation of bystander CPR								
Yes	489/4686	(10.4%)	1.72	(1.50––1.97)	<0.001	1.38	(1.14–1.66)	0.001
No	405/6367	(6.4%)	ref.			ref.		
Shock by a public‐access AED								
Yes	136/259	(52.5%)	14.64	(11.35–18.89)	<0.001	2.85	(2.06–3.95)	<0.001
No	758/10 794	(7.0%)	ref.			ref.		
Time from collapse to contact with patient by EMS (1‐min increment)	…		0.92	(0.91–0.94)	<0.001	0.91	(0.89–0.93)	<0.001
Year (1‐y increment)	…		1.05	(1.02–1.08)	0.003	1.07	(1.03–1.11)	<0.001

AED indicates automated external defibrillator; CI, confidence interval; CPR, cardiopulmonary resuscitation; EMS, emergency medical service; OHCA, out‐of‐hospital cardiac arrest; OR, odds ratio; Ref., reference; VF, ventricular fibrillation.

a The outcome information of 6 patients was missing.

## Discussion

This study revealed that the proportion of patients with exercise‐related OHCA receiving bystander‐initiated CPR and PAD considerably increased over the 8‐year study period and was associated with a significant improvement in patient survival. Because exercise‐related OHCA is more likely to be a witnessed event and associated with ventricular fibrillation,^6^ widespread dissemination of PAD programs—emphasizing the implementation of bystander‐initiated CPR as well as AED use to achieve rapid defibrillation^18^, ^19^, ^20^—should be effective in improving patient survival. In Osaka Prefecture, the number of the persons in the general population receiving any form of CPR training increased from ≈112 000 to 170 000 during the study period.^21^ Furthermore, since the implementation of AED deployment in public locations in July 2004, the cumulative number of public‐access AEDs increased, reaching >25 000 devices in 2013.^9^ Our findings confirmed that the PAD programs in Osaka Prefecture were effective in improving outcomes of exercise‐related patients with OHCA.

In the present study, ≈40% of exercise‐related OHCAs occurred in sports facilities; this was lower than the incidence reported previously (ranging from 52% to 58%).^6^, ^22^, ^23^, ^24^ Sports facilities have been identified as being particularly suitable locations for the implementation of a PAD program.^25^, ^26^ A retrospective analysis reported higher survival rates among patients sustaining OHCA at exercise sites where AEDs were available than among patients sustaining OHCA in non–exercise‐related indoor sites.^7^ Our results also showed that patients sustaining OHCA in sports facilities had a higher chance of survival than patients sustaining OHCA in other locations. The Japanese Circulation Society guidelines^27^ recommend placing public‐access AEDs in locations where the risk of OHCA occurrence is high, and these include sports facilities. Our recent research also revealed that the proportion of public‐access AED pad application to patients with OHCA was high in sports facilities.^28^ The accessibility of AEDs in sports facilities, possibly in combination with the presence of well‐trained staff, may have resulted in good patient outcomes following OHCA. This study, however, found that a considerable proportion of OHCAs occurred outside sports facilities; therefore, there is room for improvement in public AED deployment to enable rapid defibrillation in different settings. A potential strategy would be the continued expansion of AED placement in locations where people tend to exercise, such as athletic fields, schools, public parks, and popular jogging trails. Furthermore, public education to increase awareness also has an important role in improving bystander CPR and PAD.

Patients with bystander‐witnessed, exercise‐related OHCA were substantially more likely to survive with a neurologically favorable outcome compared with patients sustaining non–exercise‐related OHCA (44.1% versus 7.5%). This finding could be explained by a number of well‐established prognostic factors for OHCA, such as a shockable rhythm on presentation, younger age, better ADLs before OHCA, bystander CPR and PAD, and shorter EMS response time. Nevertheless, multivariate analysis showed that even after adjusting for these variables, exercise‐related OHCA was still significantly associated with better neurological outcome. This finding suggests the existence of other exercise‐related variables that may have a positive impact on resuscitation outcomes. A previous study in The Netherlands reported similar results, and the authors suggested that improved survival in patients with exercise‐related OHCA was related to the cardioprotective effects of regular exercise and the higher arousal status of the sympathetic nervous system.^29^

Consistent with previous reports,^6^, ^22^, ^29^, ^30^, ^31^ the majority of exercise‐related OHCAs among the general population were observed in men. The reasons for these differences between the sexes could not be determined from our data and may result from variations between men and women in exercise participation rates and duration or level of exertion during each exercise session. Sex‐related differences in the prevalence of underlying causes and later onset of coronary heart disease in women may also explain this finding,^32^, ^33^ as male sex may be an independent risk factor for cardiac arrest.

The present study demonstrated that the incidence of exercise‐related OHCA in Osaka Prefecture was low (3.1 per million population during the overall study period), representing a small subset of the overall OHCA burden (0.7% of all OHCA patients). Reports indicate that the incidence rate of exercise‐related OHCA among the general population varies according to study location: 22 (per million population per year) in the United States, 21 in The Netherlands, 6 in England, 5 in France, and 1 in Germany.^6^, ^22^, ^29^, ^30^, ^31^ These figures, however, cannot be directly compared with those in our study, owing to differences in participant ethnic distribution, exercise participation rates, target age range, study period, methodology for selecting the study population, and definition of exercise‐related OHCA. Although there is no standard definition of exercise‐related cardiac arrest, most studies have focused on cardiac arrests occurring during exercise or up to 1 hour following cessation of physical activity.^34^, ^35^ In the Utstein Osaka Project, however, patient activities at the time of arrest (exercise‐related or non–exercise‐related) were evaluated by the EMS personnel in charge on the basis of an interview with bystanders at the scene. Consequently, the exercise‐related OHCAs in our study were limited to cardiac arrests occurring during exercise. Previous studies estimated the proportion of cardiac arrests occurring during sports activities as ranging from 66% to 92% of all exercise‐related arrests;^6^, ^22^, ^30^, ^31^ therefore, the exercise‐related OHCA incidence rate in our study may be an underestimation.

### Limitations

This study has several inherent limitations. First, we did not obtain information on several factors that could affect OHCA occurrence and outcome in this Utstein Style registry. This information would include the type of exercise that patients participated in at the time of cardiac arrest, exercise intensity, frequency of habitual exercise, and details of dissemination of public‐access AEDs in the study area, as well as past medical history, medication, and other life habits. Second, our study area was limited to a single densely populated area in Japan consisting of multiple metropolitan centers; therefore, results may not be generalizable to other areas worldwide. Considering that the popularity of specific sports, the population composition, and other environmental circumstances vary in different communities or nations, the pattern of OHCA occurrence could also differ; therefore, further investigations using data from other communities worldwide are needed to confirm our findings. Finally, data uncertainty, validity, integrity, and ascertainment bias were also possible sources of bias in this study. The classification of presumed cardiac arrests, for example, was determined by exclusion diagnosis; therefore, the specific cause of the arrest could not be determined with certainty. Nevertheless, the large sample size, covering all OHCA patients in Osaka Prefecture, and the adoption of the standardized data format based on the Utstein Style reporting guidelines^13^, ^14^ minimized potential bias.

## Conclusions

In recent years, the rates of exercise‐related OHCA in the target population have increased, although the incidence remained low overall. The significant increases in CPR and use of public‐access AED defibrillation by bystanders were associated with substantial improvement in patient outcome after exercise‐related OHCA.

## Sources of Funding

This study was supported by a scientific research grant from the Ministry of Education, Culture, Sports, Science and Technology of Japan (16K09034).

## Disclosures

None.
